# Case Report: *Candida dubliniensis* as a Cause of Chronic Meningitis

**DOI:** 10.3389/fneur.2020.601242

**Published:** 2020-12-08

**Authors:** Madiha Tahir, Andrew M. Peseski, Stephen J. Jordan

**Affiliations:** ^1^The University of Vermont Health Network-Champlain Valley Physicians Hospital, Plattsburgh, NY, United States; ^2^Division of Internal Medicine, Department of Medicine, Indiana University School of Medicine, Indianapolis, IN, United States; ^3^Division of Infectious Diseases, Department of Medicine, Indiana University School of Medicine, Indianapolis, IN, United States

**Keywords:** *Candida dubliniensis*, chronic meningitis, fungal meningitis, case report, intravenous drug use, hepatitis C

## Abstract

**Background:** *Candida dubliniensis* is closely related to *Candida albicans* and rarely isolated in clinical specimens. *C. dubliniensis* is increasingly recognized as a pathogen in immunocompromised hosts. We present the third known case of *Candida dubliniensis* meningitis in a young immunocompetent host.

**Case Presentation:** A 27-year-old female with a history of intravenous heroin use and chronic hepatitis C presented with a 10-month history of headaches and progressive bilateral vision loss. On physical examination, visual acuity was 20/20 in her right eye and grade II papilledema was noted. Examination of her left eye revealed complete loss of vision and grade IV papilledema. An MRI with and without contrast revealed increased leptomeningeal enhancement involving the posterior fossa and spinal cord. After multiple lumbar punctures, cerebrospinal fluid fungal cultures grew *Candida dubliniensis*. The patient was successfully treated with a combination of liposomal amphotericin and fluconazole for 6 weeks with complete resolution of her CNS symptoms, with the exception of irreversible vision loss.

**Conclusion:** We report a case of chronic meningitis due to *Candida dubliniensis* in an immunocompetent woman with hepatitis C and a history of intravenous heroin use. Additional studies are needed to confirm risk factors for *Candida dubliniensis* colonization, which likely predisposes individuals to invasive candidiasis.

## Introduction

*Candida dubliniensis* is a dimorphic yeast that was first described in 1995 in the oral cavity of HIV-infected individuals (1). Though phenotypically similar to *C. albicans, C. dubliniensis* is thought to be less virulent due to decreased expression of phospholipases, aspartyl proteinases and virulence genes (2–4). *C. dubliniensis* colonizes the oropharynx and respiratory tract of immunocompromised hosts and is increasingly being recognized as an opportunistic pathogen in blood, soft tissue, urogenital, gastrointestinal and most recently, corneal compartments (5, 6). However, risk factors for *C. dubliniensis* colonization are poorly studied. *C. dubliniensis* meningitis is exceedingly rare, especially in immunocompetent individuals (7–11). We present the third known case of *Candida dubliniensis* meningitis in a young immunocompetent host and suggest that heroin use may be a risk factor for *C. dubliniensis* colonization.

## Case Description

A 27-year-old female with a history of intravenous heroin use and chronic hepatitis C presented to the emergency department with a 10-month history of headaches and progressive bilateral vision loss. She last injected heroin 10 months prior, just before her symptoms began. She described her headaches as constant, bi-frontal, and throbbing in nature without any exacerbating or alleviating factors. She reported associated nausea with occasional vomiting, a 40-lb weight loss, and associated cervical and thoracic back pain. Additionally, she denied fevers, tinnitus, loss of consciousness, weakness, or seizures. Within the past 2 months, she developed progressive bilateral vision loss (left greater than right) and presented to an ophthalmology clinic on the day of admission, where she was diagnosed with papilledema and referred to the emergency department.

On physical examination, the patient appeared well. Her temperature was 98.3°F (36.8°C), pulse was 90 beats per min, blood pressure was 113/65 mmHg, respirations were 16 breaths per min, and her oxygen saturation was 97% on room air. Her pupils were dilated at 4 mm bilaterally. Visual acuity was 20/20 in her right eye and grade II papilledema was noted. Examination of her left eye revealed complete loss of vision and grade IV papilledema. The rest of her physical examination was unremarkable, including no evidence of neck rigidity and negative Kerning's and Brudzinski's signs. She refused a jolt accentuation test.

**Figure 1:**
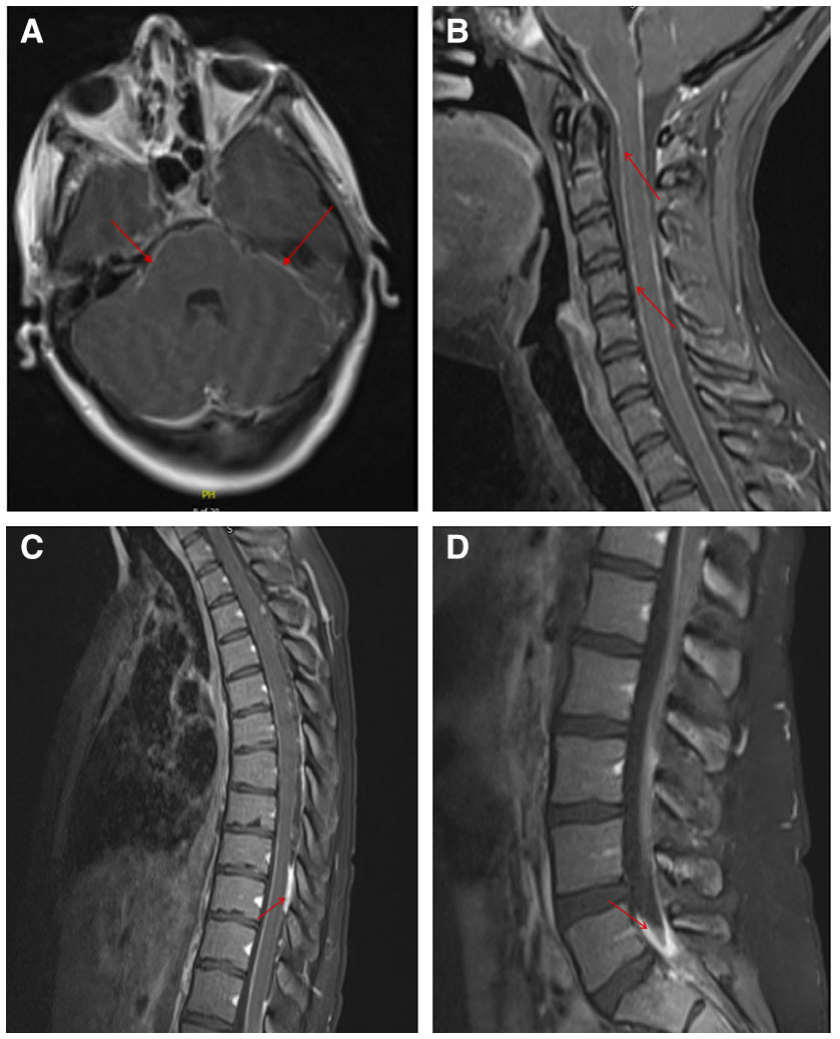
Leptomeningeal enhancement demonstrated on MRI with and without contrast of the brain and spinal cord. MRI brain axial image showing leptomeningeal enhancement of the posterior fossa and the visualized proximal spinal cord **(A)**. Leptomeningeal enhancement was noted in sagittal images of the cervical spine **(B)**, thoracic spine **(C)**, and lumbar spine **(D)**. Areas of hyperintensity are denoted by the red arrows.

To further evaluate for infectious meningitis, a lumbar puncture was performed, which showed an opening pressure of 35 cm H_2_O. Cerebrospinal fluid (CSF) analysis revealed clear, colorless fluid, glucose of 14 mg/dL (reference range 40–70 mg/dL), a total white blood cell count of 730/mm^3^ (reference range 0–10/mm^3^) with a differential of 58% neutrophils, 30% lymphocytes and 12% monocytes, a red blood cell count of 8.0/mm^3^ (reference range 0.0–5.0/mm^3^), and protein level of 251 mg/dL (reference range 15.0–46.0 mg/dL). Additional CSF testing from the initial lumbar puncture was negative for the following tests: VDRL, flow cytometry for lymphoma, cryptococcal antigen, acid-fast bacteria (AFB) smear, GeneXpert® MTB/RIF PCR, Biofire® Filmarray® meningitis/encephalitis multiplex PCR and Gram stain without organisms present. A comprehensive metabolic panel and complete blood count with differential were normal, with the exception of an elevated aspartate transaminase of 106 IU/L (reference range 15–37 IU/L) and alanine transaminase of 152 IU/L (reference range 12–78 IU/L). Initial imaging with an MRI venogram of the head was negative for dural venous thrombosis. An MRI brain with and without contrast revealed increased leptomeningeal enhancement involving the posterior fossa and visualized proximal spinal cord (Figure 1A), concerning for tuberculosis, neurosarcoidosis, or CNS carcinomatosis. Given these findings, the patient was admitted to the hospital for treatment of suspected bacterial meningitis and infectious diseases was consulted.

The patient was empirically started on vancomycin and ceftriaxone, but antibiotics were stopped when CSF cultures remained negative at 48 hours. The patient admitted to prior incarceration and living in homeless shelters but denied *Mycobacterium tuberculosis* (TB) exposures. Given known TB risk factors, hypoglycorrhachia with a partial lymphocytic pleocytosis, and confirmed leptomeningeal enhancement evidenced on MRI, she was empirically started on rifampin, isoniazid, pyrazinamide, and ethambutol for potential tuberculous meningitis while additional TB tests were pending. Given her reported thoracic back pain, a subsequent MRI of her total spine with and without contrast was performed which showed diffuse leptomeningeal enhancement of the cervical (Figure 1B), thoracic (Figure 1C) and lumbar spinal cord (Figure 1D), including the cauda equina, distal pons, medulla and basal cisterns. Further, enhancing intradural extramedullary nodules at the level of T3, T7 and T10 were seen within the cord.

To further narrow the differential diagnosis, additional serum testing was performed which showed normal ACE levels, negative HIV-1/2 serologies, a negative RPR, and a negative T-spot. A repeat lumbar puncture was performed on day 5 to obtain additional CSF for repeat GeneXpert® MTB/RIF testing and CSF cultures. On hospitalization day 11, a CSF fungal culture from the second lumbar puncture grew *Candida dubliniensis*. A 3rd lumbar puncture was performed to repeat the CSF fungal culture, which again grew *C. dubliniensis*, and to obtain a CSF Beta-(1,3)-D-glucan level, which was above the limit of detection >500 pg/mL, (reference range <60 pg/mL). A diagnosis was made of *C. dubliniensis* meningitis.

Upon confirmation of the diagnosis of *C. dubliniensis* meningitis, tuberculosis treatment was stopped and intravenous amphotericin B lipid complex (5 mg/kg/day) was initiated. After 14 days of amphotericin, a repeat lumbar puncture was performed and a CSF fungal culture was negative; the CSF beta-D-glucan level had decreased to 36 pg/mL (reference range <60 pg/mL). The patient was then discharged with 4 weeks of oral fluconazole 800 mg daily to complete 6 weeks of treatment. The patient was seen 2 weeks post-discharge in the infectious diseases clinic and reported adherence to treatment and complete resolution of her headache, neck and back pain; her vision remained unchanged.

## Discussion

Chronic meningitis can be a diagnostic dilemma and delays in correct diagnosis and treatment may lead to worse neurologic outcomes (12). *Candida* species, including *Candida dubliniensis*, are a rare cause of infectious meningitis and are associated with chronic meningitis (7). Risk factors for invasive candidiasis include neurosurgical interventions, previous treatment with antibiotics, parenteral nutrition, abdominal surgery, and an immunocompromised state such as HIV/AIDS, malignancy, or chronic steroid use (13, 14). Increasingly, non-*C. albicans* etiologies of meningitis have been reported, like *C. tropicalis, C. parapsilosis*, and *C. glabrata* (14–18).

In this case report, we describe the sixth known case of *C. dubliniensis* meningitis—the third known case in an immunocompetent host—and suggest that intravenous drug use may be a risk factor for *C. dubliniensis* colonization. In invasive candidiasis, including candidal meningitis, species identification and azole resistance testing are critical in order to identify the optimal antifungal therapy as antifungal resistance profiles vary by *Candida* species. Although specific *Candida* species are associated with unique risk factors [*C. tropicalis* with head and neck surgery (18), *C. parapsilosis* with medical devices and catheters (17), *C. glabrata* with the urogenital tract (19), etc.], diagnosing *Candida* as a cause of chronic meningitis requires a high level of suspicion in the setting of negative CSF bacterial cultures. Given no sign or symptom has adequate specificity to distinguish candidal meningitis from other causes, diagnosis requires isolating *Candida* from CSF. As the sensitivity of CSF culture (the gold standard diagnostic test) varies widely by causative organism, repeat lumbar punctures and other diagnostic testing may be necessary as prior evidence suggests that the organism burden may be lower in chronic meningitis (20). Sensitivity can be improved by large volume taps (10–20 mL) or the use of adjunct tests, like the Beta-(1,3)-D-glucan (BDG) assay. Though the BDG test is FDA approved for serological diagnosis of invasive fungal disease, it is not yet approved for CSF; however, a cutoff of ≥80 pg/mL has been demonstrated to have a sensitivity and specificity of 96 and 95%, respectively, for diagnosing invasive fungal meningitis (21). In our study, we were unable to isolate C. *dubliniensis* on the initial CSF culture and multiple subsequent cultures were needed. Even though CSF BDG is not specific for a diagnosis of Candida meningitis, a markedly elevated level (>500 pg/mL) in our case was highly supportive of the diagnosis. CSF BDG levels may be used to support a diagnosis of invasive fungal meningitis if there is a high clinical suspicion while additional CSF cultures are obtained to confirm the diagnosis.

Why *C. dubliniensis* infection of the CNS may have caused 10 months of slowly progressive neurological symptoms in our patient is unclear. Although *C. dubliniensis* is phenotypically similar to *C. albicans* and both are the only *Candida* species that can produce hyphae and chlamydospores (4), several observations suggest that it may be less virulent than *C. albicans*. The increased virulence in *C. albicans* is thought to be due to its ability to tolerate thermal and oxidative stress (4).

We hypothesize that intravenous heroin use was the risk factor for *C. dubliniensis* colonization in our patient. In patients with HIV/AIDS, *C. dubliniensis* is primarily associated with asymptomatic oral colonization, which is likely the risk factor for developing invasive candidiasis. However, *C. dubliniensis* is an uncommon oral commensal in immunocompetent individuals and rarely causes fungemia or invasive disease (4). Although *C. dubliniensis* meningitis has rarely been reported, cases of *C. dubliniensis* fungemia are increasingly being reported and suggested risk factors include indwelling catheters, recent broad-spectrum antibiotics, end-stage liver disease, IV drug use, and immunocompromised states including HIV, recent chemotherapy and solid organ transplant (7). Of the previously reported five patients with *C. dubliniensis* meningitis (Table 1), four had a confirmed history of intravenous drug use and drug use in the remaining two was unknown; two appeared immunocompetent and of similar age to our patient (7–11). Therefore, the most commonly identifiable risk factor for *C. dubliniensis* meningitis appears to be intravenous drug use. Indeed, this would support a prior study of 60 patients that demonstrated a history of drug use increased the frequency of colonization with non-*albicans* species, especially *C. dubliniensis* (22). Whether these individuals shared needles with immunocompromised individuals that had oral colonization of *C. dubliniensis* or engaged in associated behaviors that predisposed to oral colonization (e.g., poor oral hygiene) is unknown. Other reported risk factors for *C. dubliniensis* meningitis in prior cases were hepatitis C-related cirrhosis, insulin dependent diabetes mellitus and lung transplant recipient (7, 10). These risk factors likely resulted in transient fungemia with hematogenous spread to the CNS. Indeed, one patient had *C. dubliniensis* fungemia prior to developing meningitis (10). Although our patient did not have documented fungemia, she reported injecting heroin just prior to the onset of her CNS symptoms and hematogenous spread to the CNS may have occurred. Additional similarities between our case and others is that 1) diffuse leptomeningeal enhancement seems to be a common finding and may be helpful in evaluating chronic meningitis; and 2) multiple lumbar punctures may be necessary to isolate *C. dubliniensis* (8, 10, 11).

**Table 1:** Comparison of clinical characteristics and management of six known cases of *C. dubliniensis* meningitis.

	**Van Hal et al. (10)**	**Andrew et al. (11)**	**Yamahiro et al. (7)**	**Wilson et al. (8)**	**Herrera et al. (9)**	**Tahir et al. (2020)**
Gender	Male	Male	Male	Female	Male	Female
Age (years)	48	25	49	26	74	27
History of hepatitis C	Unknown	No	Yes	Unknown	Yes (in lung donor)	Yes
History of intravenous drug use	Unknown	Yes	Yes	Yes	Unknown	Yes
Immunocompromised	Yes (bilateral lung/heart transplant)	No	Yes (cirrhosis)	Unknown	Yes (lung transplant)	No
Leptomeningeal enhancement on MRI	Present	Present	Present	Present	Absent	Present
Prior *C. dubliniensis* Fungemia	Present	Absent	Absent	Absent	Absent	Unknown
Treatment	Caspofungin followed by fluconazole	Liposomal Amphotericin B and oral flucytosine followed by oral fluconazole	Liposomal Amphotericin B and intravenous flucytosine followed by fluconazole (intravenous and then oral)	Unknown	First course: voriconazole followed by oral fluconazole Second course:Liposomal Amphotericin B followed by oral voriconazole	First course: Amphotericin B, followed by oral fluconazole
Total Duration	Unknown	8 weeks	30 days	Unknown	First course: 8 weeks Second course: Unknown	6 weeks
Clinical Outcome	Survived	Survived	Expired	Survived	Survived	Survived

In our patient, we report successful clearance of *C. dubliniensis* from the CSF with 2 weeks of amphotericin B followed by 4 weeks of high-dose oral fluconazole; the isolate was confirmed to be sensitive to azoles. Given the limited number of reported cases, the optimal treatment for *C. dubliniensis* meningitis has yet to be defined. In the previous five cases, treatment involved various combinations of antifungal agents; specific agent, treatment duration, and outcomes for each case can be seen in Table 1 (7–11). Of the five cases, one did not survive despite treatment possibly due to delay in diagnosis and treatment. Our patient had complete resolution of her CNS symptoms, with the exception of irreversible vision loss.

## Conclusion

*Candida dubliniensis* is a rare cause of chronic meningitis, especially in immunocompetent hosts. Intravenous drug use may be a risk factor for *C. dubliniensis* colonization and subsequent invasive candidiasis. Hence, in intravenous drug users, clinicians should maintain an index of suspicion for *C. dubliniensis* fungemia and hematogenous spread to the CNS in absence of other identifiable causes of chronic meningitis.

## Data Availability Statement

The original contributions generated for this study are included in the article/supplementary material, further inquiries can be directed to the corresponding author/s.

## Ethics Statement

Written informed consent was obtained from the individual for the publication of any potentially identifiable images or data included in this article.

## Author Contributions

MT and AP wrote the initial draft. MT, AP, and SJ reviewed and approved the final manuscript. SJ overviewed case and final manuscript.

## Conflict of Interest

The authors declare that the research was conducted in the absence of any commercial or financial relationships that could be construed as a potential conflict of interest.
